# Naturally Occurring Polyelectrolytes and Their Use for the Development of Complex-Based Mucoadhesive Drug Delivery Systems: An Overview

**DOI:** 10.3390/polym13142241

**Published:** 2021-07-08

**Authors:** Raúl Cazorla-Luna, Araceli Martín-Illana, Fernando Notario-Pérez, Roberto Ruiz-Caro, María-Dolores Veiga

**Affiliations:** Departamento de Farmacia Galénica y Tecnología Alimentaria, Facultad de Farmacia, Universidad Complutense de Madrid, 28040 Madrid, Spain; racazorl@ucm.es (R.C.-L.); aracelimartin@ucm.es (A.M.-I.); fnotar01@ucm.es (F.N.-P.); rruizcar@ucm.es (R.R.-C.)

**Keywords:** naturally occurring polyelectrolyte, mucoadhesion, polyelectrolyte complex, drug delivery systems, polyelectrolyte multilayers

## Abstract

Biopolymers have several advantages for the development of drug delivery systems, since they are biocompatible, biodegradable and easy to obtain from renewable resources. However, their most notable advantage may be their ability to adhere to biological tissues. Many of these biopolymers have ionized forms, known as polyelectrolytes. When combined, polyelectrolytes with opposite charges spontaneously form polyelectrolyte complexes or multilayers, which have great functional versatility. Although only one natural polycation—chitosan has been widely explored until now, it has been combined with many natural polyanions such as pectin, alginate and xanthan gum, among others. These polyelectrolyte complexes have been used to develop multiple mucoadhesive dosage forms such as hydrogels, tablets, microparticles, and films, which have demonstrated extraordinary potential to administer drugs by the ocular, nasal, buccal, oral, and vaginal routes, improving both local and systemic treatments. The advantages observed for these formulations include the increased bioavailability or residence time of the formulation in the administration zone, and the avoidance of invasive administration routes, leading to greater therapeutic compliance.

## 1. Introduction

Naturally occurring polymers can be obtained from renewable natural sources such as animals, plants, or fungi. These biopolymers can be divided into proteins, such as albumin and zein, and polysaccharides, including chitosan and pectin [1]. Interest in biomacromolecules for medical applications has grown in recent years as they are nontoxic, non-immunogenic and can be degraded by in vivo enzymes. Their metabolites have low toxicity to organisms, and they are also capable of controlled drug release due to their swelling capacity. Another advantage is that these polymers can generally be chemically modified thank to the moieties on the chains of these biomacromolecules, which gives them a great versatility for medical objectives [1].

Many biopolymer-based healthcare materials have recently been explored, such as drug delivery designs; some of them have already been approved for clinical use, and biopolymers are widely employed as excipients in the pharmaceutical industry [2]. Furthermore, most of the natural polymers are mucoadhesive, which makes them interesting candidates for the development of drug delivery systems intended for the sustained release of drugs in the different mucous membranes (i.e., ocular, nasal, buccal or vaginal) [3]. Moreover, biopolymers have proven capable of biomimicking the native extracellular matrix and a judicious choice of the processing method may enhance this feature. Accordingly, different techniques, such as electrospinning or 3D printing, are presently being explored for the manufacture of biopolymer-based healthcare materials, including wound dressings or scaffolds for tissue engineering [4,5,6]. Furthermore, biopolymers have been used as coating agents in tissue engineering with numerous advantages, such as the improvement of osteogenic expression or the inhibition of biofilm formation while providing anti-inflammatory effects. Interestingly enough, these coatings can be loaded with drugs and nanoparticles, acting as drug delivery systems [7]. Pectin or chitosan are some representatives of this application for biopolymers [8,9].

However, the use of biopolymers in tissue engineering or as drug carriers is complicated due to their broad molecular weight distributions, their batch-to-batch variability and their short shelf-life [5,10,11]. To overcome these drawbacks, the introduction of functional moieties onto the polymer backbone is a widely studied strategy. For instance, reactive groups of chitosan allow different derivatives to be obtained, such as acetylated chitosan, alkylated chitosan or carboxylated chitosan, among others. These modifications lead to alterations in the physicochemical characteristics that can be exploited for the development of healthcare materials. Although to a lesser extent, other biopolymers have been modified to the same end, such as xanthan gum, guar gum or pectin [12,13,14]. Nonetheless, biopolymer derivatives that arouse the most interest in the field are thiomers, also known as thiolated polymers, which are obtained through the immobilization of sulfhydryl-bearing molecules onto the polymer chain [15]. Both anionic and cationic biopolymers can be thiolated, including chitosan, alginate or pectin [13,16,17,18]. Biopolymer-based thiomers are known to be promising polymer derivatives, as there are different properties that can be improved compared to the native polymer, such as mucoadhesion or mechanical properties. These features are valued for the development of drug delivery systems, as they can result in sustained drug releases and longer residence times in the release zone [19]. On the other hand, natural polymers can be combined into polyelectrolyte complexes (PECs), which are the result of the interaction of an anionic and a cationic polymer. These structures also make it possible to optimize the properties of biopolymers, which is why they are increasingly being investigated in the development of healthcare materials [20].

In this manuscript, (i) the main characteristics of the most commonly used naturally occurring polyelectrolytes are detailed, (ii) the main properties, methods of manufacturing and applications of biopolymer-based polyelectrolyte complexes based on natural polymers are described and (iii) the scientific literature based on the use of polyelectrolyte complexes for the manufacture of mucoadhesive drug delivery systems is reviewed.

## 2. Naturally Occurring Polyelectrolytes

Naturally occurring polyelectrolytes (PEs) are linear or branched biopolymers in which a substantial proportion of the constituent units are ionizable or ionic groups. These macromolecules can therefore dissociate on dissolving in a polar solvent such as water, resulting in the charge of the backbone and generating a polyion. The charge on the repeating units of the PE is neutralized by oppositely charged smaller counterions which preserve the electroneutrality, so after dissociation, these counterions are released into the medium. The polymer chains are generally observed to extend upon dissociation, due to the repulsion between electrostatic forces. Adding a counterion to the medium (in the form of a salt) leads to the screening of the electrostatic interactions, and hence to the contraction of the polymer coils [21,22].

PEs are neutral macromolecules in their native form but have unique properties after dissociation due to their electrostatic interactions, which translates into great potential for their application in the field of drug delivery [20,23]. These properties include particularly their water solubility, ionic conductivity, strong intrachain and interchain interactions, interaction with ions (small molecules or PEs), and surface activity [24]. With this backdrop, various combinations of PEs have been explored: oppositely charged PEs, PE–micelles, PE–protein or oppositely charged block copolymers [25].

Both polycations and polyanions can mucoadhere through different mechanisms. Mucoadhesion in polycations, which are protonated at physiological pH, takes place mainly by electrostatic interactions with mucin, negatively charged due to the presence of sialic acid. Although electrostatic repulsion could be expected in the case of anionic polymers, Van der Waals, hydrogen bonds and hydrophobic interactions all play a vital role in the mucoadhesion process [26].

### 2.1. Classification

PEs can be classified into different groups:

1. Depending on the ionizable groups:
  - Polyanions
  - Polycations
  - Polyampholytes
    - Annealed
    - Quenched
    - Betainic
2. Depending on the dissociation ability:
  - Strong
  - Weak
3. Depending on the charge location:
  - Integral
  - Pendant
4. Depending on the composition:
  - Homopolymers
  - Heteropolymers or copolymers
5. Depending on the origin:
  - Natural (biopolymers or biopolyelectrolytes)
  - Semisynthetic
  - Synthetic

The main division is based on their ionizable groups. These groups can be either acidic–commonly known as polyacid or polyanion—or basic—called polybase or polycation. If the macromolecule contains both acidic and basic groups along their backbone, these PEs are called polyampholytes [23], which can be sorted into three classes: annealed, quenched and betainic (also known as zwitterionic). If the acidic and basic monomers are ionized depending on the pH of the medium, the polyampholyte is described as annealed. If charged cationic and anionic monomers retain their respective charges independently of the pH, the polyampholyte is classified as quenched. Betainic—or zwitterionic—polyampholytes contain identical numbers of fully charged anionic and cationic groups in the same monomer units. These macromolecules therefore compensate the cationic–anionic monomer pairs, without the need for counterions [27].

PEs dissociate in aqueous media under appropriate conditions determined by each PE. PEs can be classified into strong or weak depending on their degree of dissociation in aqueous media. Strong PEs generally dissociate independently of the pH of the medium, whereas the dissociation of weak PEs is strongly conditioned by the pH [23].

Another interesting classification is based on the location of the charge; if the charged groups are integrated into the backbone chain, the PE is classified as integral; and if the charged groups are attached as side groups, the PE is described as pendant [23].

According to the characteristics of the molecule, if the macromolecule is composed of only one type of repetitive unit (or monomer), it is called homopolyelectrolyte; and if two or more monomers form the backbone of the PE, it is classified as heteropolyelectrolyte or copolyelectrolyte [20].

Lastly, PEs can be divided according to their origin as either natural PEs (biopolymers or biopolyelectrolytes, such as proteins or polysaccharides), semisynthetic PEs (such as carboxymethylcellulose) or synthetic PEs (such as sulfonated polystyrene) [24].

### 2.2. Biopolycations

#### Chitosan

Among the industrially relevant natural polysaccharides, chitosan is the only high molecular weight cationic biopolyelectrolyte; the other naturally occurring polysaccharides for industrial uses are either neutral or anionic [28,29].

**Figure 1:**
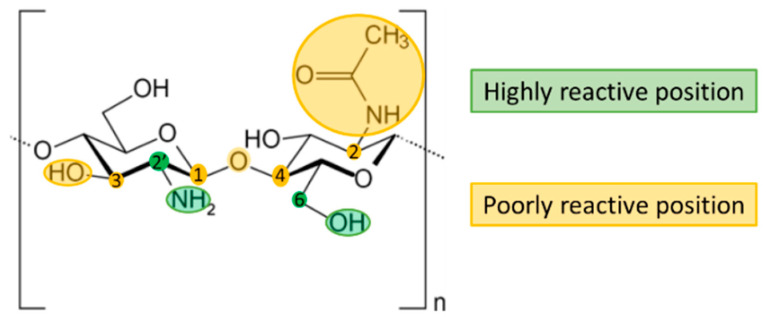
Chemical structure of chitosan. Highly reactive positions are highlighted in green, poorly reactive position are highlighted in yellow.

Chitosan, composed of glucosamine and N-acetyl-glucosamine, is the name given to a group of naturally occurring polysaccharides which can be directly extracted from various fungi [30], although it is usually produced by the alkaline deacetylation of chitin obtained from the exoskeleton of crustaceans [31]. A low degree of deacetylation leads to an increase in the solubility and viscosity of the gel formed, while a high molecular weight can decrease the solubility and increase the viscosity of chitosan [32]. The chemical structure of chitosan is shown in Figure 1. The chemical properties of C6-OH and C2′-NH2 can be exploited to introduce other functional groups, which can improve its physical and chemical properties, and broaden its applications and relevant fields of research [33]. 

Chitosan has the unique feature of adhering to mucosal surfaces thanks to the presence of hydroxyl and amino groups in its structure, which can interact with the mucus through electrostatic interactions and hydrogen bonds [34]. Owing to its nontoxic, biodegradable and biocompatible properties, chitosan has already been approved by FDA for use in wound dressings. Chitosan-based drug delivery systems have aroused great interest since the early 1990s, and several works have reported on chitosan and its potential application in biomedical fields, including wound dressing, tissue engineering and therapeutic drug delivery.

Chitosan is insoluble in both water and organic solvents but can dissolve in aqueous solutions of acids due to the presence of amine groups in its structure. The protonation of the polymer chains produces a polycation, which implies that a higher deacetylation leads to an increase in free amino groups and therefore to higher solubility in acidic media [35]. Although acetic acid is frequently used for chitosan solubilization, other acids such as lactic acid, tartaric acid and citric acid are currently being explored. These acids have good organoleptic properties compared to acetic acid, whose taste and odor can induce rejection for biomedical applications. The acid used for the solubilization of chitosan also modifies its characteristics in terms of its rheology, pH and film-forming properties [36]. 

### 2.3. Biopolyanions

Most natural polysaccharides are neutral or negatively charged, and there are multiple references in the literature to the use of biopolyanions to form PECs [37]. This review will focus on those that have been used to develop mucoadhesive dosage forms, but it should be noted that any other biopolymers could be used in the future for the development of PEC-based mucoadhesive systems.

#### 2.3.1. Alginate

Alginate is the name given to the natural polysaccharides commonly extracted from three species of brown algae including *Laminaria hyperborean*, *Ascophyllum nodosum*, *Macrocystis pyrifera* and *Saccharina japonica*, but it can also be isolated from *Azotobacter vinelandii* and several *Pseudomonas* species such as *Pseudomonas aeruginosa* and *Pseudomonas mendocina* [38,39]. This polymer represents up to 40% of the dry weight of these algae, and is found in seaweeds neutralized with counterions such as magnesium, strontium, barium and sodium [40]; alginates can also be found commercially in the form of sodium, potassium or ammonium salts [41].

**Figure 2:**
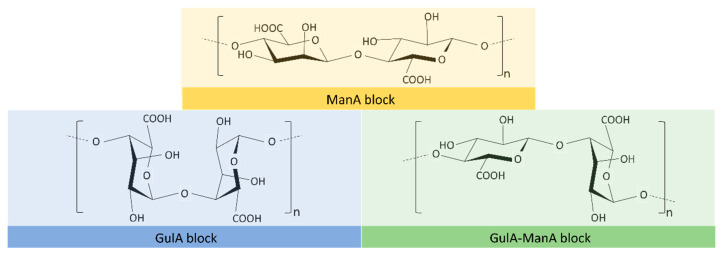
Chemical structure of the different blocks that can be found in alginate.

Alginate is an unbranched polymer composed of (1 → 4)-linked β-D-mannuronic acid (ManA) and α-L-guluronic acid (GulA), which have a pKa of 3.65 and 3.35, respectively. It is, therefore, negatively charged across a wide range of pH, displaying chain homosequences (GulA blocks and ManA blocks) interspersed with heterosequences (GulA-ManA blocks) (Figure 2), with molecular weights between 60 KDa and 700 KDa [41,42]. 

Alginate can form a gel independently of the temperature, mainly via two methods, although other procedures are being explored:

1. Acid gelation: Alginic acid gels are obtained when the pH of the medium is lower than the constant of dissociation (or pKa) of the polymer. A rapid decrease in pH implies the precipitation of polymeric aggregates, while a progressive drop in pH leads to the formation of a continuous gel. Acid gels from alginate are stabilized by hydrogen bonding, with residues of ManA blocks playing a major role in the gelation process. Gel strength is known to be correlated with the content of GulA blocks in the alginate chain [41].
2. Ionotropic gelation: Alginate can form ionic gels in the presence of multivalent cations, which are being widely explored for biomedical applications such as drug encapsulation and cell immobilization. Alginate affinity towards cations is directly dependent on the number of G-blocks present in the alginate structure, and increases in the order of Mn < Zn, Ni, Co < Fe < Ca < Sr < Ba < Cd < Cu < Pb. It should be noted that some of these cations are toxic, and cannot be considered for biomedical application; Pb, Cu, and Cd exhibit high toxicity. Calcium alginate gels, which are highly biocompatible, are the most commonly used for the development of biomedical devices. The gelation of alginate occurs through the binding of divalent cations and the GulA blocks by dimerization of GulA residues. Thus, Ca ions cause the two GulA blocks to bind on opposite sides, forming a diamond-shaped hole consisting of a hydrophilic cavity that binds the Ca ions to the oxygen atoms of the carboxylic groups. This tightly bound polymer conformation has been described as an egg-box-like structure, where each cation binds with four G residues in the egg-box formation to form a 3-D network. The binding of trivalent cations with alginate is generally enhanced compared to divalent cations, as they are able to interact with three carboxyl groups from different alginate biopolymers at the same time, forming a three-dimensional bonding structure that produces a more compact network [41].
3. Non-conventional methods: Other methods worth highlighting are cation-free cryogelation, ionotropic cryogelation, non-solvent induced phase separation and carbon dioxide induced gelation, among others [43].

The mucoadhesive character of alginate may enhance its usefulness as a potential vehicle for prebiotic and probiotic bacteria and drug delivery in mucosal tissues. Studies have shown that polymers with charge density can serve as good mucoadhesive agents; hence alginate, which is an anionic polymer thanks to its carboxylic groups, is a good mucoadhesive biopolymer. Alginate’s capacity to adhere to mucosal surfaces allows microorganisms or drugs to be retained on these surfaces, thus improving the effectiveness of probiotics or drugs [39]. Alginate has also been studied for tissue engineering thanks to some of its properties such as porosity, mechanical strength, cell proliferation, excellent mineralization and osteogenic differentiation [44].

#### 2.3.2. Pectin

**Figure 3:**
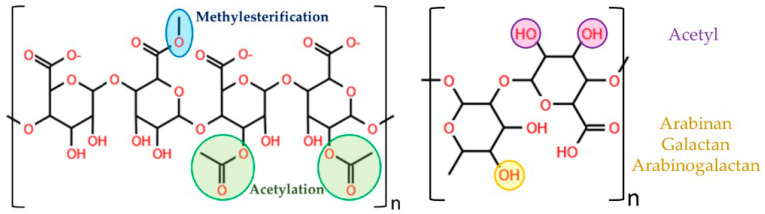
Basic structure of the homogalacturonan (left) and rhamnogalacturonan-I (right) backbones. In homogalacturonan, the carboxyl groups in C-6 are partially methylesterified (blue). The hydroxyl groups in C-2 and C-3 are partially acetylated (green). In rhamnogalacturonan-I, Rhap residues are substituted at C-4, indicated in yellow. The backbone residues of GalpA may be O-acetylated on C-2 and/or C-3, indicated in purple.

Pectins are weak polyanionic heteropolysaccharides found in the cell walls of land plants, and are frequently obtained from fruit such as apple or citrus peel. They are also present in green algae. Structurally, three pectic polysaccharides have been isolated from primary cell walls. Homogalacturonan is composed of a chain of α-(1→4)-D-galacturonic acid (GalpA) (≥65%). The carboxyl groups in C-6 can be partially methylesterified and the free acid groups may be partly or fully neutralized with sodium, potassium or ammonium ions. Pectins may also be O-acetylated on C-2 or C-3 [45,46]. Rhamnogalacutonan-I is a backbone of GalpA and rhamnopyranosyl (Rhap) in the repeating dissacharide [→4)α-D-GalpA-(1,2)-α-L-Rhap-(1→] which represents 20–35% of pectin. The backbone residues of GalpA may be O-acetylated on C-2 and/or C-3. Rhap residues are substituted at C-4 with neutral and acidic oligosaccharide side chains such as arabinose and galactose, forming arabinan, galactan and arabinogalactans in the side chains. Other less frequent residues may also be present, as well as ferulic or coumaric acid (Figure 3) [46,47,48]. Finally, rhamnogalacturonan-II is the most structurally complex pectic domain, and represents 10% of pectin. Its structure is widely conserved across plant species and consists of a highly branched homogalacturonan backbone. The side chains contain 11 rare sugars in over 20 different linkages. Rhamnogalacturonan-II is usually found in plant walls as homodimers crosslinked by a 1:2 boratediol ester [48,49].

Pectins are classified according to their degree of esterification (also known as degree of methoxylation) according to the proportion of carboxyl groups esterified with methyl groups, which is directly related to the gelling mechanism and, in general, with the properties of the polymer. Pectins are considered high methoxyl if more than 50% of the carboxyl groups are esterified, and low methoxyl if this figure less than 50% [45,46].

As an ionic branched macromolecule with high molecular weight, pectin has interesting properties for drug delivery, such as its mucoadhesiveness and the ability to dissolve in basic environments and to form gels in acidic media. This polymer is generally considered biocompatible, nontoxic and biodegradable, and a good option for the development of drug delivery systems or tissue engineering [1]. The abundant carboxyl groups on the pectin chains can ionically cross-link with calcium ions to form an “egg-box” structure, and the proportion of calcium ions in the pectinate calcium structure conditions the process of drug release [50]. 

#### 2.3.3. Xanthan Gum

**Figure 4:**
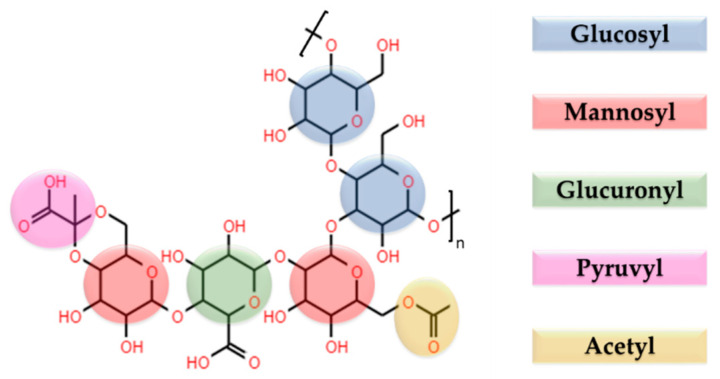
Schematic representation of the chemical structure of xanthan gum.

Xanthan gum is a highly hydrophilic natural heteropolysaccharide produced by the bacteria *Xanthomonas campestris*. It is a branched polymer with high molecular weight in the range of 2000 KDa to 20,000 KDa. Its primary structure is a repetitive pentasaccharide: the backbone is a linear (1 → 4)‒linked‒D‒glucose, with a trisaccharidic side chain containing varying proportions of acetyl and/or pyruvyl on the mannose residues, with a varying degree of substitution (Figure 4). Due to the pyruvyl residues, xanthan gum is a natural anionic PE with a pKa = 3.1, which makes it a suitable candidate for the development of PEC-based devices [51,52,53].

It has been demonstrated that xanthan gum takes a helical secondary structure in aqueous medium, which undergoes an “order-disorder” transformation from helix to coil structure depending on several factors (i.e., pH, ionic strength, nature of the ions in the medium, acetyl and pyruvyl contents). The secondary structure can be obtained by high temperatures and low salt concentrations; the rigid ordered state at low temperature becomes more flexible in the disordered state when the temperature rises, producing a conformational transition of the solution. Salts stabilize the order conformation, which is responsible for the extraordinary stability of this biopolymer. Xanthan gum chains interact with bivalent cations to form three-dimensional networks. Low pyruvyl content is known to lead to low viscosity, while high pyruvate content promotes gel behavior through macromolecular association; high acetyl content hinders the gelation of the polysaccharide in aqueous solution [52,54,55].

Xanthan gum solutions are formed in aqueous medium in two steps; first, water penetrates the polysaccharide, which imbibes it and swells. The xanthan gum macromolecules then diffuse and dissolve in the medium, so the polymer dissolves slowly. The solution formed is known to have a non-Newtonian rheology, with a shear-thinning behavior under an increasing shear rate. It has a proven ability to form a highly viscous solution at low shear forces even at low concentrations with high pseudo-plasticity. Xanthan gum solutions are stable over a wide range of temperatures and pH, up to 90 °C and a pH between 2 and 11. Xanthan gum solutions also exhibit little change in viscosity, depending on the salinity, and have a high resistance to mechanical degradation [52,56,57].

Another feature of xanthan gum is its great mucoadhesivity, thanks to the large number of hydroxyl groups that can form hydrogen bonds with other moieties, enabling the polymer to interact with the mucin on the mucosal surface. Xanthan gum has been reported to exhibit a better mucoadhesion performance than other known highly mucoadhesive polymers such as tragacanth gum, chitosan and hydroxypropylmethylcellulose [58,59].

#### 2.3.4. Gum Arabic

Gum arabic is a complex polysaccharide exuded from acacia trees (mainly *Acacia Senegal* and *Acacia seyal*) composed of three distinct fractions with different protein contents and molecular weights: the arabinogalactan fraction, the arabinogalactan–protein fraction and the glycoprotein fraction [60]. Arabinogalactan is the major component (≈88 wt.%) and consists of a highly branched β-(1 → 3)-galactose backbone with sidechains composed of L-arabinose, L-rhamnose, D-glucuronic acid and 4-O-methyl-D-glucuronic acid (MW = 300 KDa). Due to the two last residues, it is negatively charged above pH 2.2 [61,62]. Arabinogalactan protein, a smaller fraction (10 wt.%), is a high molecular weight arabinogalactan–protein complex (MW = 1000–1500 KDa). The protein represents 10 wt.% of the fraction [60,62]. Glycoprotein is the smallest fraction(1–2%wt), and is also an arabinogalactan–protein complex, but with 50 wt.% protein content [62].

Gum arabic is known to exhibit a wide variation in the molecular weight distributions of the fraction, conditioned by the origin, species and age of the tree. Processing conditions may also modify the composition of the gum. A variety of molecular mass values have been observed for gums exuded from different parts of the same tree, implying an inter- and intra-tree variation [63].

One of the most important properties of gum arabic is its function as a highly efficient emulsifier and long-term stabilizer in products containing oil–water interfaces. The arabinogalactan–protein complex appears to be responsible for these properties, stabilizing the interface in the oil-droplets. However, the adsorption affinity of this polymer is low, which explains the high concentration of gum required to efficiently cover the oil–water interface and achieve effective emulsification and stabilization [61].

Gum arabic solutions have been found to have very low viscosities, due to the fact that compared to other biopolymers, the highly branched structure of gum arabic has low intermolecular interactions and does not generate a three-dimensional structure [64]. Hydrogels based on gum arabic therefore have limited mechanical and rheological properties, and are often brittle [65]. 

#### 2.3.5. Carrageenan

**Figure 5:**
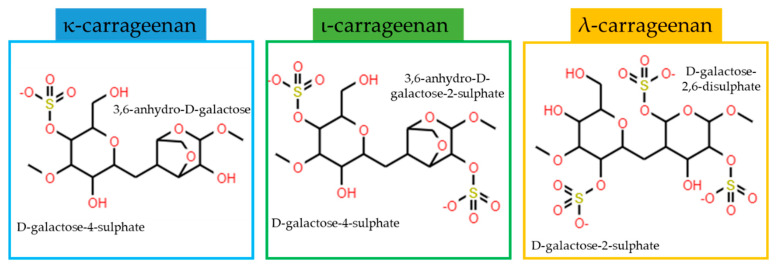
Chemical structures of the most industrially relevant carrageenans.

Carrageenan is a group of sulphated natural polyanions obtained from red algae. The interest in these polymers lies in their structural diversity and unique physical properties. They are water-soluble linear polysaccharides composed of repeating disaccharide units with alternating 3-linked β−D-galactopyranose and 4-linked α-galactopyranose or 3,6-anhydro-α-galactopyranose with an average molecular weight of between 100 and 1000 KDa. The structural diversity of CG is due to the location and percentage of the sulphate groups (15–40%) or the presence of 3,6-anhydro-D-galactose [66,67]. At least 15 different carrageenan structures are known today, with varying chemical structures and degrees of sulphation. However, three types of carrageenan are most important to industry: κappa (κ), ιota (ι) and lambda (λ) carrageenan (Figure 5) [68]. 

κ and ι types are generally obtained from algae of the *Eucheuma* and *Kappaphycus* genera, and λ type is extracted from the family *Gigantinaceae* [69]. *Ι*-carrageenan has proved able to form brittle gels, while λ-carrageenan forms softer paste-like gels. In aqueous solutions, both forms undergo a reversible conformational arrangement at higher temperatures, and a network formation through sulphate groups and 3,6-anhydro-D-galactopyranosyl rings at lower temperatures. They can also gel in the presence of cations, a process that is influenced by the polymer and cation concentrations. The nature of the cation also affects the gelation process; for instance, the formation of stronger κ-carrageenan gels has been confirmed in the presence of KCl as compared to other salts, while for ι-carrageenan, the storage modulus is rapidly increased with a divalent salt concentration but slowly with a monovalent salt concentration. It has been proposed that carrageenan chains form gels via coil-to-helix transition, after which these helices aggregate in parallel with the sulphate groups towards the outside; this structure is stabilized by hydrogen bonds. Unlike the other two forms described, λ-carrageenan does not have the ability to thermogel, but can gel using trivalent cations [67,70].

Carrageenans are widely used as emulsifiers and gelling, thickening and stabilizing agents in pharmaceutics and food technology. They also have inflammatory; immunomodulatory and anticoagulant properties, anticancer activity, and immunomodulatory, anti-hyperlipidaemic and antioxidant properties. They exert a protective activity against bacteria and fungi and inhibit some viruses such as herpes and papillomavirus. However, carrageenans are mainly used as excipients in drug delivery, tissue engineering and regenerative medicine [69,71].

#### 2.3.6. Hyaluronic Acid

**Figure 6:**
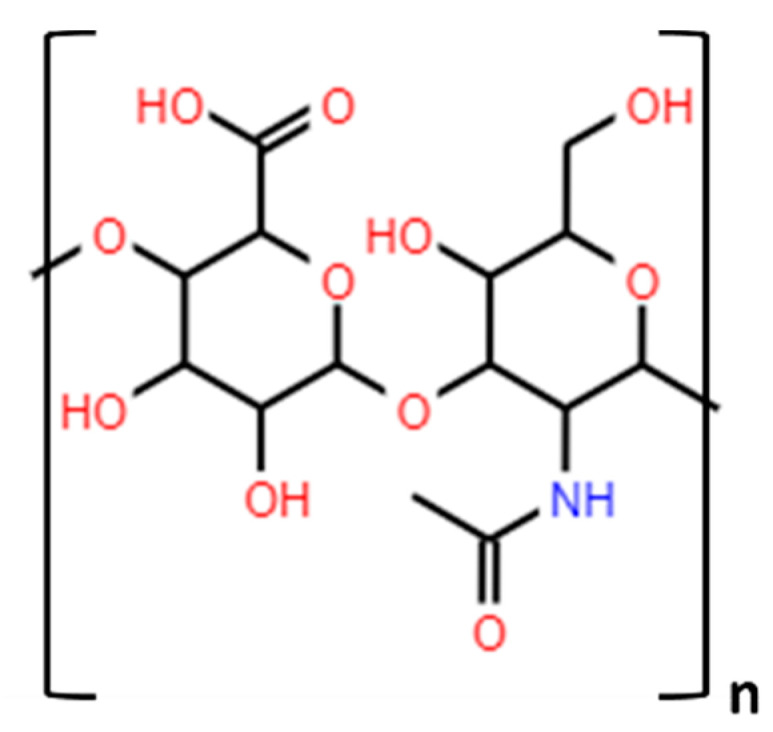
Chemical structure of hyaluronic acid.

Hyaluronic acid is an anionic biopolymer, a major component of the extracellular matrix present in the connecting tissues of all vertebrates [72]. Presently, it is frequently obtained from natural sources such as bovine vitreous, shark skin, rooster combs, and numerous microorganisms [73]. Its chemical structure is a linear glycosaminoglycan composed of repeating units of N-acetyl-D-glucosamine and D-glucuronic acid, linked via alternating β-(1 → 3) and β-(1 → 4) glycosidic bonds (Figure 6) [74]. It can be found in a wide range of molecular masses (from 100 to 8000 KDa) [75]. 

At physiological pH, hyaluronic acid is negatively charged, and highly hydrophilic, so intra- and intermolecular hydrogen bonds are formed in aqueous media. This prevents the rotation of the molecule which causes the chains to have a rigid position, so hyaluronic acid gels show high viscosity and viscoelasticity. It has been reported that these parameters increase as the molecular weight of the polymer rises, whereas a rise in temperature or the addition of electrolytes can increase the mobility of the chains by reducing the viscosity in solution [73].

Hyaluronic acid is biocompatible, biodegradable, non-inflammatory, nontoxic and non-immunogenic, which is why it has aroused interest in various fields of medicine, such as drug delivery, visco-supplementation, eye surgery and bone-tissue engineering, among others [74,76]. Unfortunately, the greatest drawback of hyaluronic acid for its use in medicine is its short half-life, which limits the use of the pristine polymer, so modifications such as chemical reactions or the formation of PECs have been proposed to improve its molecular stability [37,77].

## 3. Polyelectrolyte Complexes

PECs form through an electrostatic interaction between the polymer cation and the polymer anion when they are mixed in a solvent, where they spontaneously interact and form the complex. It has been demonstrated that the formation of the complex is largely driven by electrostatic interaction between the counter polyions, but also involves hydrogen bonding and Van der Waals interactions [78,79]. Although electrostatic charge compensation produces the ordering of two oppositely charged polyions to a complex molecule with conformational changes favorable for both counter polyanions, the chaotic aggregation of polyanions and polycations also seems to occur, with only partial mutual charge compensation. Several ionic sites are therefore still charge-compensated by low molecular weight counterions [80].

**Figure 7:**
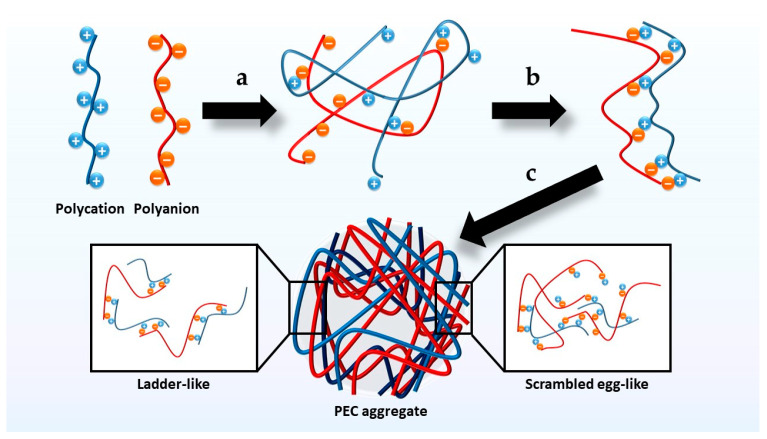
Diagram of the phases in the formation of polyelectrolyte complexes (PECs). (**a**) Establishment of secondary binding forces. (**b**) Formation of new bonds and correction of the distortions of the polymer chains. (**c**) Aggregation of the complexes formed through hydrophobic interactions.

The mechanism of PEC formation can be summarized in three main steps: the first, which occurs immediately, is the establishment of secondary binding forces after mixing oppositely charged PE solutions; the second involves the formation of new bonds and/or the correction of the distortions of the polymer chains, leading to a new conformation; and the last is the aggregation of the complexes formed through hydrophobic interactions, which are generally insoluble in the medium [80,81]. It has been suggested that the complexation process may result in a ladder-like or scrambled-egg-like PEC. Ladder-like PECs are formed by hydrophilic single-stranded and hydrophobic double-stranded parts. These phenomena are the result of mixing PEs with weak ionic groups and large differences in the size of their molecules, which produces higher viscosity and an electromagnetic shielding effect compared to the constituent PEs. In contrast, due to precipitation by aggregation, scrambled-egg-like PECs exhibit lower viscosity than their constituent PEs [81,82]. However, considering their mechanism of formation, PECs can be regarded as a combination of both with different ratios depending on the characteristics of the polyions [80] (Figure 7).

The interaction between the polycation and the polyanion usually produces a macroscopic phase separation, and the resulting complex is accompanied by structural and dynamic features. Strong electrostatic attractions usually lead to the formation of a solid precipitate, whereas weak pairs of PEs are more likely to form coacervates [83]. However, some PECs have proved capable of forming a turbid colloid or even dissolving in the medium when the PEs are small [81]. The formation and properties of the PECs depend on factors such as the molecular weight, ionic strength, hydrophobicity, charge density, concentration and chain rigidity of the polymers and the pH of the medium, among others. 

Due to their versatility, PECs are useful for increasing the structural, mechanical and thermal properties of PEs, which can enhance the performance of the devices (such as allowing longer drug release times or improving the resistance of scaffolds for tissue engineering) and facilitate manufacturing processes by improving the mechanical characteristics (increasing tablets’ resistance to fracture or allowing the formation of fibers by electrospinning) [21,82,84]. The pH-dependent ionization of the constituent PEs can be leveraged to design PECs whose assembly and disassembly capacity is pH-dependent, and used to develop smart drug delivery systems, for example [36,78]. Another possibility is the use of PECs for drug vectorization and targeting thanks to their ability to bind and penetrate cells through different types of interaction, particularly electrostatic interactions [81]. PECs have therefore attracted considerable interest for their importance in basic research, but also in biological and technological applications.

### 3.1. Polyelectrolyte Complex-Based System Production Methods

PEC-based hydrogel production methods vary depending on the polymers used. In general, hydrogels containing either the polyanion or polycation are prepared separately, ensuring the pH of the medium allows the formation of PEs. For instance, acetic acid is commonly used to ensure the protonation of chitosan. Once both hydrogels are obtained, they are blended and the PEC-based hydrogel is formed [85,86]. However, the combination of biopolymers in a tablet for in situ gelation has also been previously reported; after hydrating in an aqueous medium, the hydrogel forms spontaneously [84]. PEC-based hydrogels can be further processed to obtain other systems such as inserts by freeze-drying or films by the solvent casting method [87,88]. On the other hand, the combination of polyanions and polycations leads to the formation of insoluble polydispersions under specific conditions, which leads to the formation of PEC-based nanoparticles. Some of this conditions are the location and strength of the ionic sites, the presence of precursor chemicals, the pH or environmental factors such as temperature or stirring intensity [81].

**Figure 8:**
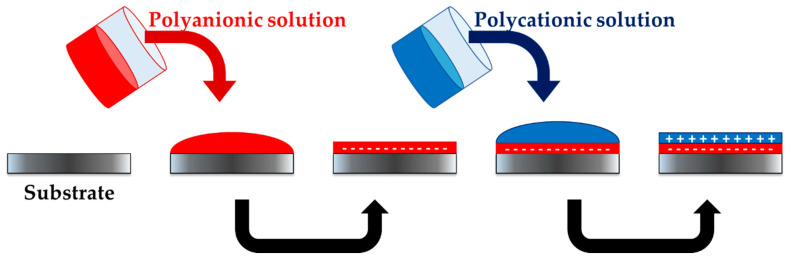
Process for obtaining polyelectrolyte multilayers. A solution containing a polyion is poured onto a substrate, which forms a polyionic layer. A solution containing a counter polyion is then poured onto the previously formed layers, thus forming a PEM.

Layer-by-layer (LbL) is a method for preparing highly tunable thin multilayer polymer films, known as polyelectrolyte multilayers (PEMs). As in the case of PECs, PEMs self-assemble by electrostatic interactions between sequentially deposited alternately charged PE films (Figure 8). The properties of PEMs can be optimized by controlling several parameters such as the pH or ionic strength of the PEMs, the number of layers or the order in which the layers are deposited [89]. This simple and versatile technique produces not only thin films but also coatings, even in substrates with complex surfaces. Additionally, this technique rarely requires organic solvents or extreme processing conditions, making LbL an attractive technique for biological-based applications [90]. It has therefore been widely studied in recent years and applied to thin film coatings, micropatterning, nanobioreactors, artificial cells, drug delivery systems, and even electronic devices. Multilayered films can also act as drug protective reservoirs; APIs can be embedded in the multilayer, thus preserving their bioactivity and increasing the stability of drugs up to the time of administration. However, the field of practical clinical applications remains to be explored [90,91].

### 3.2. Properties of Biopolymer-Based PECs

The advantages of biopolymers, such as their mucoadhesion and high swelling capacity due to water uptake, have been widely explored. These properties can be improved by forming PECs that combine two biopolymers, as the formation of PECs produces several modifications in the physicochemical properties of the system compared to the separate constituent PEs, and these properties can be decisive in the performance of several medical devices.

**Figure 9:**
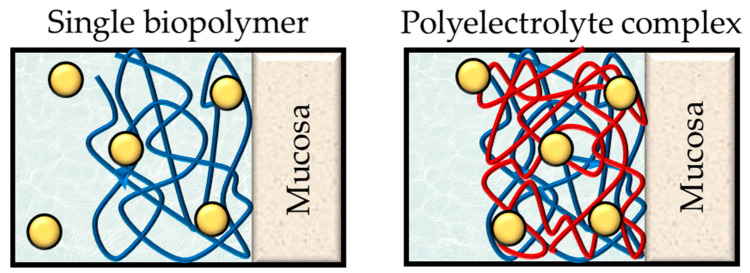
Swollen drug delivery systems based on a single biopolymer and on a polyelectrolyte complex. After the polymer swells, the medium is interposed between the polymer chains. The strong interaction between the polyelectrolytes prevents the separation of the polymer chains, and the high cross-linking between the chains means a greater capacity to control drug release and adhere to mucosa.

Biopolymers are known to entrap large amounts of water within their structure, in a phenomenon known as swelling. When the biopolymer chains are combined by electrostatic interactions, this combination has higher resistance to the penetration of aqueous media and moderate swelling ratios compared to the biopolymers separately; it has been confirmed that the water uptake of PECs is not equal to the sum of the swelling behavior of the constituent PEs [84]. Swelling is directly related to most of the properties of PECs, and the swelling of the PECs can be a determining factor in the performance of the formulations. Drug release subsequently occurs more slowly due to the entanglement of the compact structure generated after immersing the drug delivery system in an aqueous medium [84]. Interestingly, it has also been observed that low swelling ratios lead to more efficient mucoadhesion (Figure 9) [59].

Mucoadhesion can be described as the ability of many polymers to bind the molecules of the mucus layer. Although it is hard to discern whether the interaction occurs on the cell surface or between the molecules of the polymer and the mucus layer [92], five theories are commonly presented to explain this phenomenon. Although none of these theories alone can explain mucoadhesion, several can be combined to describe the process [93,94]:

1. Electronic theory: electron transfer occurs upon contact between the polymer and the mucus surface due to different electronic charges in their structure. This leads to the adhesion of the surfaces through the formation of an electrical double layer at the interface.
2. Adsorption theory: the polymer binds the mucus by weak chemical interactions, for instance Van der Waals forces or hydrogen bonds, or hydrophobic interactions.
3. Wetting theory: this refers to the polymer’s ability to spontaneously spread over the mucus surface and develop adhesion.
4. Diffusion theory: a process driven by concentration gradients and affected by the available molecular chain lengths and their mobilities. Sufficient depth of penetration creates a semi-permanent adhesive bond, and this depends on several parameters such as the nature of the mucoadhesive chains, the diffusion coefficient and the flexibility and motility of the polymer chains.
5. Mechanical theory: according to this theory, a liquid adhesiveness is created with the presence of irregularities in the mucosal surface, as this increases the area of contact between the polymer and the mucosa.

**Figure 10:**
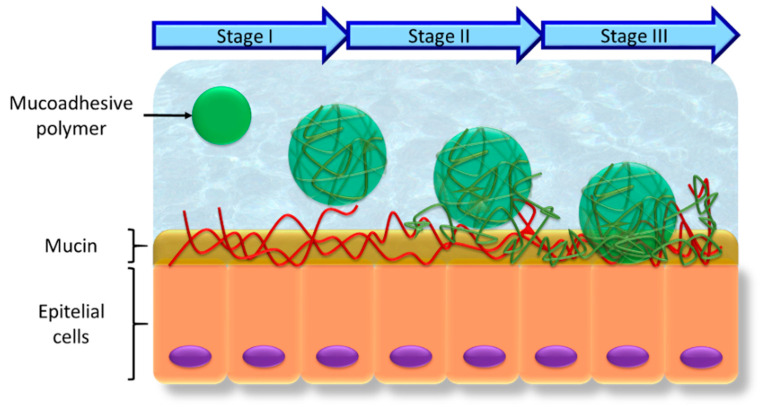
Diagram of the stages of mucoadhesion. I is the contact stage. During stage II, the polymer chains and the mucosal surface interpenetrate. In stage III, covalent bonds are formed between the entangled polymer chains and the mucosal surface.

In view of the different mechanisms described, the mucoadhesion process can be divided into three different steps (Figure 10) [94,95]:

1. Contact stage: the first stage occurs when the biopolymer is wetted in the medium and swells after it is placed on the mucous membrane. This is governed by the wetting theory.
2. Polymer chains and mucosal surface interpenetration: the polymer chains of the biopolymer and the mucosal layer become entangled by forming physical bonds. This occurs through the combination of the adsorption theory and electronic theory, and is favored by the mechanical theory.
3. Creation of bonds between the chains or consolidation stage: the entangled polymer chains, bound by physical interaction, consolidate the adhesion by forming covalent bonds. This is governed by the combination of the diffusion theory, electronic theory and adsorption theory.

The mucoadhesiveness of the biomacromolecules can be improved through the formation of PEC, as the high entanglement between both PEs hinders the penetration of water. This can be attributed to three features: first, the mucoadhesive groups of both biomacromolecules are combined within the same systems; second, the system maintains its structure so does not spread, but eventually leaks; and third, the mucoadhesive groups have greater density as the polymer chains are less separated [96]. Finally, mucoadhesion should be considered a characteristic of the system as a whole, so the presence of different functional groups can increase the interaction between the system and the mucosa. Since there is an electrostatic interaction between the oppositely charged polymer chains, the spectra obtained by FTIR corroborates the formation of the PECs by the formation or displacement of bands. The modifications must be carefully studied depending on the constituent PEs, giving special importance to the bands that can be assigned to the ionizable groups. The three-dimensional structures formed after this electrostatic interaction differ from those seen for each of the components separately. SEM is generally used to observe these processes, and multiple references confirm the presence of unique three-dimensional structures thanks to the formation of PECs [84]; as a result, their porosities differ from the constituent polymers, which may be relevant to their functionality. This is the case of scaffolds for tissue engineering, since the systems must allow the correct diffusion of gases in order to maintain a moist environment and enhance cell attachment, proliferation and differentiation [97,98]. It is also important in the case of drug delivery systems, since the pore size can condition the drug delivery process [99]. Porosity studies can be performed by the alcohol displacement method [97,98] or through Hg porosimetry [84].

Lastly, some thermodynamic properties such as the enthalpies or temperatures of the phase transition are frequently modified after the electrostatic interaction of PEs for the formation the PECs [100,101]. Thermogravimetry (TGA) and differential scanning calorimetry (DSC) have also been applied to the characterization of PECs. For instance, the thermal degradation determined through TGA is usually modified and the DSC data generally show an offset shift in the peaks observed in the PEs separately, or new peaks—both endothermic and exothermic—may even appear [98,102].

Other more specific techniques can be found in the literature, i.e., rheological analysis [103], ζ-potential [104], mucoadhesion studies [84], and X-ray diffractometry [102], among others.

### 3.3. Polyelectrolyte Complexes for Medical Applications

**Figure 11:**
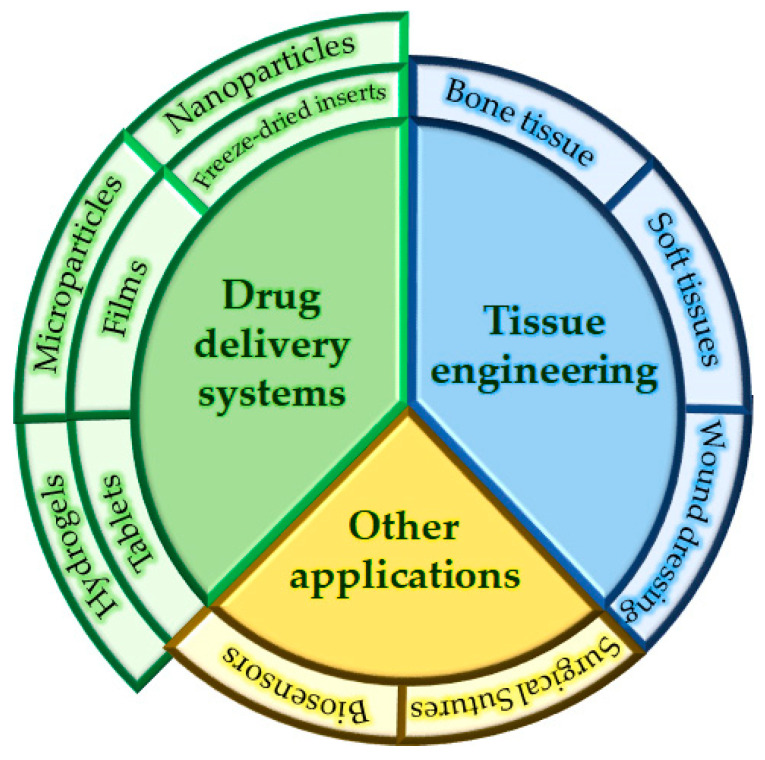
Diagram of the main applications found in the literature for PECs.

Due to chitosan’s exclusive ability to form a polycation, it forms PECs with polyanions [37], including particularly biopolyanions; alginate, pectin, xanthan gum, gum arabic and carrageenan are the most frequently used PEs [100,105]. However, semisynthetic polymers such as carboxymethylcellulose [106] and synthetic polymers such as methacrylic acid derivatives [107] have also been used. PECs based on polysaccharides and their derivatives have aroused great interest for their medical application; compared with chemically crosslinked polymer complexes, PECs based on biopolymers—especially polysaccharides—are generally considered to be nontoxic and biocompatible, and therefore of potential interest for the design of medical devices. Several biopolyelectrolyte-based PECs have been shown to have various applications in medical and pharmaceutical areas, and particularly in drug delivery systems, tissue engineering and wound dressing (Figure 11).

#### 3.3.1. Drug Delivery Systems

Many PECs have been proposed for the controlled release of drugs, as they can be very useful for sustaining drug release or preserving the properties of sensitive biological molecules [108]. According to Meka et al., there are four mechanisms for preparing drug-loaded PECs [20]:

1. Dissolution of the drug in the medium, followed by its entrapment in the precipitation of the complex.
2. Absorption of a dissolved drug in the preformed PEC (especially with sponge-like PECs).
3. Chemical binding of the drug to polyanions or polycations, and the subsequent formation and precipitation of the PEC.
4. Use of the drug as a partner in the formation of the PEC. This requires the drug to possess at least one ionizable polar group.

Although PECs have the advantage of being biocompatible and therefore nontoxic, their use as carriers for drugs or nanoparticles can lead to cytotoxicity issues. In addition to the fact that nanoparticles can be cytotoxic depending on several properties (such as size, morphology or surface charge density), it is well known that they undergo biodegradation in the cellular environment and degraded products could accumulate within the cells, inducing severe damage. According to this, a thorough understanding of the kinetics and toxicology of the particles is needed and cytotoxicity studies are therefore frequent and necessary for PEC-based drug delivery systems containing nanoparticles [109,110]. However, in vitro studies are limited and do not provide adequate prediction of the effects that the administration of the dosage form will have; this highlights the need for in vivo studies.

Table 1 shows a list of different PEC-based drug delivery systems found in the literature.

Hu et al. [85] developed salecan/chitosan PEC-based hydrogels for the oral administration of vitamin C. Thanks to the properties of the PECs, the carrier allowed this highly unstable drug to be preserved in the gastric environment. The PEC also enabled a pH-dependent release, reducing the release of vitamin C in simulated gastric acid and enhancing its release in simulated intestinal fluid, so high blood levels of the drug could be maintained for at least 6 h. The hydrogel exhibited high cytocompatibility and was biodegradable. PEC-based hydrogels were also explored by Hanna and Saad [111], who prepared them by combining carboxymethyl xanthan gum and N-trimethyl chitosan for the release of ciprofloxacin as model drug. The drug was effectively encapsulated in the hydrogel and showed high activity against Gram-positive and Gram-negative bacterial strains due to the successful release of ciprofloxacin from the hydrogel matrix, even improving on the effectiveness of the reference antibiotic, gentamicin.

Syed et al. [112] prepared tablets with the aim of obtaining a controlled release of isosorbide mononitrate, which is poorly absorbed from the upper gastrointestinal tract. The tablets were prepared by combining chitosan with different polyanions (sodium alginate, xanthan gum and guar gum), and after optimizing the proportions, the chitosan/xanthan gum PEC-based tablets were found to prolong the release of the drug for up to 12 h with a good stability profile. Moin et al. [113] also prepared tablets intended for oral administration, in this case combining xanthan gum and Eudragit^®^ E100, to improve the release of diclofenac. They performed in vitro-in vivo studies and reported that the drug release was successfully prolonged for up to 12 h in vitro, and that the in vivo pharmacodynamic profile was substantially improved compared to the free drug.

Films are innovative drug delivery systems as they have several advantages: they are small, thin, and easy and economical to manufacture, and can be administered conveniently without the need for an applicator. Additionally, compared to gels, they prevent leakage [123]. We developed bilayer films by the layer-by-layer technique for the vaginal local administration of the antiretroviral drug tenofovir. These films were prepared with chitosan derivatives (chitosan lactate, chitosan tartrate and chitosan citrate) as the polycationic layer and Eudragit^®^ S100 as the polyanionic layer, anchored by the formation of a PEC in the interface. Highly mucoadhesive formulations were obtained due to the chitosan layer, with a sustained and pH-dependent drug release thanks to the presence of the polyanion [36]. Another approach to film technology was proposed by Ghaffari et al. [115], who prepared chitosan/pectin/Eudragit^®^ RS PEC-based films by blending polymer solutions. They found that the swelling of the PEC-based films was pH-dependent (higher at high pHs), and exploited this to obtain a sigmoidal pH-dependent release of a model drug, theophylline, with a slow initial release followed by a burst release induced by a modification in the pH.

In the field of micro and nano systems, Unagolla et al. [116] prepared chitosan/alginate PEC-based microparticles using the ionotropic gelation technique for the systemic delivery of vancomycin. The lyophilized PEC-based microparticles were shown to have the best control over the drug release (22 µm/day for 14 days), compared to microparticles prepared exclusively with one of the polymers. This improvement in the ability to control the release of vancomycin was attributed to the presence of a unique porous structure thanks to the formation of the PEC between chitosan and alginate. Yan et al. [118] developed PEC-based nanoparticles for oral administration, combining polycationic lactoferrin and polyanionic pectin. Those nanoparticles were used as carriers for curcumin, a potent antioxidant. This highly hydrophobic drug was successfully encapsulated in the nanoparticles, with in vitro controlled release and prominent antioxidant activity, indicating that the nanoparticles were suitable as carriers for curcumin.

PECs have also been proposed as carriers for other medical applications. For instance, they have been explored as vectors for gene delivery; Baghaei et al. [124] prepared nanoparticles containing chitosan/alginate, chitosan/hyaluronic acid and chitosan/dextran sulphate PECs. They reported that the microparticles were nontoxic after intravenous administration and had a high cellular uptake in MCF7 cell lines, and that there was a high tumor uptake of nanoparticles with a low accumulation in vital organs.

#### 3.3.2. Tissue Engineering

Another use for PECs is in tissue engineering: Florczyk et al. [125] used chitosan/hyaluronic acid PEC to manufacture porous scaffolds to mimic the tumor microenvironment, and concluded that these scaffolds can be used as an in vitro platform for the study and screening of novel cancer therapeutics. Coimbra et al. [126] prepared scaffolds of chitosan/pectin PECs for bone-tissue engineering, which induced bone-tissue proliferation with zero toxicity. Scaffolds for bone-tissue engineering can also be loaded with drugs as proposed by Sultankulov et al. [127], who prepared scaffolds with chitosan-based PECs that were also capable of releasing bone morphogenic protein 2, which improved the bone-tissue regeneration process. A similar approach was proposed by Ibrahim et al. [128], who prepared erodible sponges with xanthan gum/chitosan, polycarbophil/chitosan and Carbopol^®^/chitosan PECs loaded with rosuvastatin, which showed an increase in bone regeneration capacity in mice. Scaffolds can also be used as tools for soft tissue cell therapy, as proposed by Bushkalova et al. [129], who prepared macroporous scaffolds containing chitosan/alginate PECs and demonstrated that their structure and angiogenic potential made them interesting candidates to improve the results of mesenchymal stem cell therapy in soft tissue engineering. Lastly, PECs are also found in the literature as agents for wound dressing. Against this backdrop, Birch et al. [130] developed a hydrogel containing a chitosan/pectin PEC, and reported that this material could be used as wound dressing with an excellent exudate uptake, indicating it as a promising material for wound dressing bandages. With a similar objective, Meng et al. developed membranes of chitosan/alginate PEC that were loaded with silver sulfadiazine, which is an effective and widely used antibiotic for burn injuries in humans. These membranes could therefore be good candidates for wound dressing, as they release the drug in the damaged tissue, prevent infections and accelerate the recovery of the damaged tissue.

#### 3.3.3. Other Applications

Other innovative approaches for PECs include the development of biosensors or surgical sutures. As an example of the first, Rassas et al. [131] proposed the development of a voltametric glucose biosensor using chitosan/κ-carrageenan PEC to encapsulate glucose oxidase. This produced a more sensitive voltametric detection of glucose compared to films prepared exclusively with chitosan. In the case of surgical sutures, Mohammadi et al. [132] developed nylon monofilaments coated with chitosan/hyaluronic acid PECs. The antibacterial activity of these monofilaments (thanks to the presence of chitosan) and their ability to control the release of model drugs (Acid blue 80 and Astrazon blue F2RL) make these systems promising tools for the improvement of surgical sutures.

### 3.4. Advances in Biopolymer-Based Polyelectrolyte Complexes for Mucoadhesive Drug Delivery Systems 

Mucoadhesive systems offer several advantages in numerous routes, as shown in Table 2. 

#### 3.4.1. Ocular Drug Delivery Systems

Topical drug delivery through eye drops currently represents approximately 90% of all ophthalmic products. However, this delivery route is very inadequate; regardless of the volume administered, each eye drop is eliminated from the eye surface approximately 5 min after application, so only 1–3% of an eye drop reaches the eye tissue. This means that the amount of drug in an eye drop is much higher than required, which can produce adverse effects [133]. For this reason, multiple strategies are being explored for the development of dosage forms for the ocular route, including particularly the use of biopolymers, which have the advantage of being mucoadhesive, especially in the cornea and conjunctiva [134]. These biopolymers have other appealing properties such as biocompatibility with the eye tissues, biodegradability and mechanical strength [133].

Against this backdrop, we find several examples of PEC-based ocular drug delivery systems in the literature. For instance, Dubey et al. [135] developed brinzolamide-loaded chitosan/pectin PEC mucoadhesive nanocapsules for the treatment of glaucoma. These nanocapsules sustained the release of the drug for 8 h, which would significantly improve the release profiles of the formulations on the market. Ex vivo studies indicated that the nanocapsules enhanced the corneal permeation as they increased the residence time of the drug, with better results in reducing intraocular pressure compared to commercially available eyedrops thanks to the promising properties of the chitosan–pectin PEC. Another example in the literature is Costa et al. [136], who hypothesized that chitosan/alginate PECs in nanoparticles would sustain the release of encapsulated drugs more effectively than chitosan nanoparticles, and developed nanoparticles for ocular delivery of daptomycin. According to the results of their in vitro ocular permeability assays, around 10% of the dosage of daptomycin in the nanoparticles crossed the cell lines evaluated after 4 h of experiment, suggesting that daptomycin could be released over long periods of time. The adhesive capacity of the PEC could ensure the presence of the formulation in the ocular tissue. These results provided further evidence of the potential usefulness of biopolymer-based PECs as mucoadhesive drug delivery systems.

#### 3.4.2. Nasal Drug Delivery Systems

Intranasal drug administration has aroused great interest, as it is a non-invasive administration route for local and systemic action. The nasal route is also interesting for the possibility of directly administering drugs to the brain. The use of mucoadhesive substances avoids mucociliary clearance, thus increasing drug residence time. Although the nasal epithelium is a tight barrier, the intercellular junctional complex of the nasal mucosa is leaky, which, added to the high vascularization of the mucosa and lamina propria, provides a promising absorption surface for the drug absorption [137].

With this background, Dehghan et al. [100] developed nasal mucoadhesive inserts based on chitosan/xanthan gum PECs for the administration of promethazine as an alternative for intravenous and intramuscular administration, as although these routes exhibit good bioavailability they are invasive and may cause irritation or even severe tissue injury. These inserts allowed an efficient in vitro drug release which led to the permeation of the 90% of the drug in the first 8 h. Their bioadhesive characteristics could allow the formulation to remain in the nasal mucosa while the promethazine penetrates through the epithelium. With the same aim, Alavi et al. [87] developed a freeze-dried insert based on chitosan/κ-carrageenan PEC for the release of sumatriptan, in order to avoid the first pass effect when they are orally administered, without the need for invasive administrations such as the intramuscular route. As mentioned before, the drug could reach the area of action more easily from the nasal route. This study demonstrated that the properties of the system, including water uptake ability, mucoadhesion behavior and the drug release profile could be optimized by modifying the proportion of both polymers.

#### 3.4.3. Buccal Drug Delivery Systems

PECs have been used for the development of mucoadhesive buccal drug delivery systems for the treatment of local pathologies and for the systemic administration of drugs, thus avoiding first pass metabolism and drug degradation in the harsh gastrointestinal environment. Buccal drug absorption can also be promptly terminated in the case of toxicity by removing the dosage form from the buccal cavity [138].

As an example of buccal administration for systemic effect, the literature describes patches based on chitosan/pectin PEC for the release of carvedilol, developed by Kaur et al. [139]. These patches exhibited an increase in the bioavailability of carvedilol hydrochloride, which was 2.4 times higher compared to oral administration. The use of the PEC improved the swelling and mucoadhesive properties observed for the polymers separately. Chitosan was capable of swelling but its mucoadhesive capacity was low, whereas pectin formed a fluid gel in a few minutes, but its mucoadhesion capacity was greater than that of chitosan; the combination of both made it possible to obtain improved swelling capacity and mucoadhesion thank to the formation of the electrostatic bonds between both polymers. With regard to local buccal administration, films based on chitosan/alginate PECs have been proposed by Kilicarslan et al. [140] for the treatment of periodontitis through the local release of clindamycin. The side effects of the oral administration of antibiotics for periodontitis may cause problems in the progression of the treatment, so local application is an appropriate alternative, as it leads to a high local drug concentration and minimum adverse effects. The results of this study showed that combining alginate and chitosan at varying concentrations is a tool for the optimization of film properties. The PEC-based films obtained were able to control clindamycin release for up to 10 h and had high adhesiveness, emerging as an option for application inside the periodontal pocket. Tejada et al. [141] also developed mucoadhesive films for the local treatment of oral candidiasis by preparing miconazole-loaded mucoadhesive films containing chitosan/Carbopol^®^ 917NF, chitosan/gelatin, chitosan/gum arabic and chitosan/alginate PECs. The films based on chitosan/Carbopol^®^ 971NF and chitosan/gelatin PECs were the most suitable, and had optimal mechanical properties and mucoadhesiveness, relatively low swelling and a good drug release rate, with a higher in vitro activity against *Candida* culture than the raw drug.

#### 3.4.4. Oral Drug Delivery Systems

Mucoadhesive systems are today considered an option for improving oral drug delivery systems. Their potential advantages worth highlighting include their prolonged gastric or small intestinal residence time and the intimate contact between the delivery system and the absorption surface. Multifunctional polymers may act as permeation enhancers or enzyme inhibitors [142].

The use of mucoadhesive nanoparticles has been proposed for the administration of low-solubility or proteic drugs, as these carriers may prevent the enzymatic degradation of these substances in the harsh gastrointestinal environment [143]. With this objective, Avadi et al. [144,145] developed microparticles of chitosan/gum arabic PECs for the oral administration of insulin, thus avoiding the use of parenteral routes for the administration of this protein. According to the authors, the concentration of the polymers used in the development of the PEC allows the properties of the nanoparticles to be modulated, indicating the great versatility of the PEC. Nanoparticles are also able to enhance the permeation of insulin models through intestinal tissue, thus increasing the amount of insulin transported through the intestine compared to free insulin. Arora et al. [146] developed gastric mucoadhesive microcapsules based on chitosan/alginate PEC for the release of amoxicillin. The optimized formulation showed high drug entrapment and bioadhesive strength. The drug release was evaluated in simulated gastric fluid, and the microcapsules allowed a controlled release of amoxicillin for more than 12 h. The gastric retention of the microcapsules was assessed by in vitro mucoadhesion studies, confirming up to 8 h of residence time in the gastric environment and proving their efficacy as stomach-specific drug delivery systems for antibiotics. These microparticles were able to prevent the degradation of the drug in gastric acid, so less than 10% of the dosage they contained was degraded after 8 h. Kim et al. [147] prepared nanoparticles through ionic gelation between chitosan and gum arabic for the oral administration of quercetin. These microparticles had a higher intestinal mucoadhesion than free quercetin, leading to excellent permeation of the drug through the gastrointestinal tissue. Lastly, Boni et al. [148] prepared nanostructured chitosan/hyaluronic acid PECs for the oral administration of methotrexate, which is a highly soluble drug but one with low penetration through tissues. The nano PECs prepared proved to be highly mucoadhesive ex vivo and have low toxicity for intestinal cells. The system also allowed methotrexate to penetrate though a Caco-2 monoculture and a triple co-culture cell model.

#### 3.4.5. Vaginal Drug Delivery Systems

Multiple local infections caused by fungi, bacteria, protozoa or viruses are in the vagina, so this route has traditionally been used for the local administration of drugs. It has recently also been studied for the absorption of drugs due to its large surface area and abundant blood supply. It may be especially useful for active ingredients that undergo a high pre-systemic metabolism or which produce adverse effects in other routes, such as the gastrointestinal route. It also has the advantage of being non-invasive compared to parenteral routes (such as intravenous or intramuscular) [149,150]. Considerable progress has been made in this area of research in recent years, leading to a broad understanding of the anatomy, physiology, microflora and secretions of the vagina. Since traditional dosage forms generally suffer from leakage, messiness and a short residence time, patient compliance can be poor [3]. The main proposal to solve this problem is to design dosage forms that remain in the vaginal area for longer periods, which can be achieved by formulating mucoadhesive systems [151]. For instance, Trentor et al. [152] recently developed flexible membranes based on chitosan/alginate PECs for the release of metronidazol, which showed high stability in simulated vaginal fluid and a slow dissolution rate in this medium. They had high mucoadhesiveness and the ability to control the release of the drug over time, confirming the suitability of this PEC for prolonged treatments of vaginal infections. Abruzzo et al. [153] also developed a PEC-based vaginal drug delivery system, and prepared freeze-dried inserts containing chitosan and alginate for the vaginal administration of chlorhexidine gluconate. Their results demonstrated that by choosing the optimal ratio between the polymers it was possible to obtain a moderate hydration rate, high mucoadhesiveness and a controlled release of the drug, thus offering an interesting option for the treatment of aerobic vaginitis and candidiasis.

In the field of vaginal drug administration, we consider it especially important to highlight the efforts to develop formulations to prevent the sexual transmission of the human immunodeficiency virus (HIV). These dosage forms, known as vaginal microbicides, are considered an option for topical preexposure prophylaxis, which is a priority in the fight against HIV as directed by the Joint United Nations Programme on HIV and AIDS (UNAIDS) [96,154]. We developed tablets based on chitosan/pectin PECs for the vaginal controlled release of the anti-HIV drug tenofovir. The results demonstrated that the formation of PECs between both biopolymers in vaginal simulated fluid led to a significant improvement in the mechanical properties of the system. Consequently, the ex vivo mucoadhesion residence time was improved, as well as the rate of release of the drug compared to the biopolymers separately. These results indicated the great potential of PECs for improving the characteristics of mucoadhesive vaginal dosage forms [84].

## 4. Conclusions

Biopolymers are highly valuable tools for their application in biomedicine thanks to their high biocompatibility, the ease with which they can be obtained and their functional variety. They also have the unique property of being mucoadhesive, which makes them an essential tool for the administration of drugs in the mucosae. Since a large proportion of biopolymers are PEs, the possibilities of these substances can be multiplied through the formation of PECs, which have been shown to be very versatile physicochemical systems: their formation allows the mechanical or structural properties to be fine-tuned to obtain systems that act in a pH-dependent manner, or to improve mucoadhesion and drug release. However, PECs are a field that has yet to be explored in the development of mucoadhesive formulations for drug release; there are few references to PEC-based mucoadhesive drug delivery systems in the literature, although the results are generally very promising. Among the multiple advantages of PEC-based mucoadhesive formulations, it is worth highlighting several features: first, the possibility of systemic administration through the vaginal, buccal or nasal route of substances that would undergo strong pre-systemic metabolism if administered orally, thus avoiding parenteral routes; second, they can be useful for the prolonged administration of drugs for the effective treatment of ocular pathologies; third, PECs are also an option for oral administration of insoluble or proteic active ingredients; and last, they can be explored for the prevention of sexual transmission of HIV through the vaginal route, or the local treatment of pathologies through all these routes. However, and due to the limited research in the field of mucoadhesive PECs, their possibilities of clinical application are limited. To overcome this, future research should focus on evaluating novel drug delivery systems in animal models prior to clinical trials. In vivo studies will allow the efficacy and safety of these dosage forms to be adequately evaluated. For all the above reasons, these physicochemical systems are a field with extraordinary potential, whose knowledge could lead to very significant advances in the future for the treatment of multiple diseases.

## Figures and Tables

**Table 1:** Examples of drug delivery systems manufactured with biopolymer-based polyelectrolyte complexes.

Polyanion	Polycation	Formulation	Drug	Reference
Salecan	Chitosan	Hydrogel	Vitamin C	Hu et al. [85]
Carboxymethyl xanthan gum	N-trimethyl chitosan	Hydrogel	Ciprofloxacin	Hanna and Saad [111]
Alginate Guar gum Xanthan gum	Chitosan	Tablet	Isosorbide nitrate	Syed et al. [112]
Xanthan gum	Eudragit^®^ E100	Tablet	Diclofenac sodium	Moin et al. [113]
Kappa, Iota and Lambda carrageenan	Chitosan	Tablet	Trimetazidine hydrochloride	Li et al. [114]
Eudragit^®^ S100	Chitosan lactate Chitosan tartrate Chitosan citrate	Film	Tenofovir	Cazorla-Luna et al. [36]
Pectin	Chitosan	Film	Theophylline anhydrous	Ghaffari et al. [115]
Alginate	Chitosan	Microparticles	Vancomycin	Unagolla et al. [116]
Alginate Eudragit^®^ L100-55	Oligochitosan	Microparticles	Naproxen	Čalija et al. [117]
Pectin	Lactoferrin	Nanoparticles	Curcumin	Yan et al. [118]
Poly(maleic acid-*alt*-ethylene) Poly(maleic acid-*alt*-octadecene)	Chitosan	Nanoparticles	Methotrexate	Ciro et al. [119]
Alginate	Cationized gelatin	Nanoparticles	Curcumin	Sarika et al. [120]
Pectin	Chitosan	Nanoparticles	Insulin	Maciel et al. [121]
Alginate Xanthan gum Carbopol^®^	Chitosan	Freeze-dried inserts	Fluconazole	Darwesh et al. [122]

**Table 2:** Advantages of using mucoadhesive drug delivery systems for different administration routes.

Administration Route	Advantages of Using Mucoadhesive Drug Delivery Systems
Ocular	Reduced drug loss; less dose required and local toxicity Increased drug residence time in cornea and conjunctiva Versatile mechanical properties
Nasal	Local and systemic drug administration Increased drug residence time as mucociliary clearance is avoided Possible route for direct drug administration into the brain
Buccal	Local and systemic drug administration Reduced degradation compared to oral route; less drug degradation in intestinal medium and avoidance of pre-systemic metabolism Increased drug residence time Possibility of prompt interruption of treatment in the case of adverse reaction
Oral	Possibility of prolonged gastric or small intestinal residence time Intimate contact between the delivery system and the absorption surface Enhanced permeation Increase in drug stability and enzyme inhibition
Vaginal	Local and systemic drug administration Reduced degradation compared to oral route; less drug degradation in intestinal medium and avoidance of pre-systemic metabolism Increased drug residence time

## Data Availability

Data sharing not applicable.
